# High prevalence of *Pfmdr-1* N86 and D1246 genotypes detected among febrile malaria outpatients attending Lira Regional Referral Hospital, Northern Uganda

**DOI:** 10.1186/s13104-019-4269-1

**Published:** 2019-04-23

**Authors:** Emmanuel Achol, Stephen Ochaya, Geoffrey M. Malinga, Hillary Edema, Richard Echodu

**Affiliations:** 1 Department of Biology, Faculty of Science, Gulu University, P.O. Box 166, Gulu, Uganda; 2 Department of Immunology and Microbiology, Gulu University, P.O. Box 166, Gulu, Uganda; 3 Gulu University Bioscience Research Laboratories, P.O. Box 166, Gulu, Uganda; 4 Department of Environmental and Biological Sciences, University of Eastern Finland, P.O. Box 111, 80101 Joensuu, Finland

**Keywords:** Malaria, *Pfmdr*-1, Mutation, Drug resistance, Genotypes, Prevalence, SNPs, Resistance

## Abstract

**Objective:**

To determine the prevalence of *Plasmodium falciparum* multi-drug resistant gene-1 (*Pfmdr-1*) N86Y and D1246Y genotypes among febrile malaria outpatients attending Lira Regional Referral Hospital, Uganda.

**Results:**

Overall, 92.3% (n = 48/52) and 90% (n = 45/50) of the parasites detected carried the wild type alleles 1246D and N86, respectively. Only 7.7% (n = 4/52) and 10% (n = 5/50) of these *P. falciparum* isolates carried the *Pfmdr-1* mutant alleles 1246Y and 86Y, respectively. Our results show high prevalence of wild type alleles N86 and D1246 in *P. falciparum* isolates from Lira Regional Referral Hospital, which could translate to a decreased sensitivity to artemether-lumefantrine. Continued monitoring of prevalence of single nucleotide polymorphisms is warranted to timely inform malaria treatment policies and guidelines.

## Introduction

Malaria mainly caused by an intracellular protozoan *Plasmodium falciparum* remains a serious infectious disease with high morbidity and mortality worldwide. In 2018, the sub-Saharan Africa accounted for 92% and 93% of the global malaria cases and deaths, respectively, with4% of the total world burden occurring in Uganda [1]. Uganda has a stable and perennial malaria transmission in 90–95% of the country, with a low and unstable transmission especially in the highlands usually with potential for epidemics [2].

In the absence of effective malaria vaccines, early and successful treatment is vital in reducing morbidity and mortality. However, the emergence and spread of antimalarial drug resistance has contributed to a global increase in malaria related deaths [3]. Previously, resistance to chloroquine (CQ) and sulfadoxine–pyrimethamine (SP) hampered malaria control efforts resulting in the adoption of artemisinin-based combination therapy (ACT) as the standard treatment for uncomplicated *P. falciparum* malaria [4, 5]. ACT consists of a rapid-acting artemisinin derivative with a longer-acting partner drug that clears parasites not eliminated by the artemisinin component and limits selection of artemisinin resistance [6, 7]. The re-infecting parasites are exposed to slowly declining drug concentrations during clearance of the long-acting partner drug and a shorter post-treatment prophylactic effect could reduce the ACT to an artemisinin derivative monotherapy [8]. In 2004, artemether-lumefantrine (AL) was adopted as the first line treatment for uncomplicated malaria in Uganda, with artesunate/amodiaquine (AS/AQ) as its alternative [9].

Mutations in *P. falciparum* genes, identified as single nucleotide polymorphisms (SNPs) are markers of anti-malaria drug resistance which allow for the monitoring of declining drug susceptibility in parasite populations [10–12]. For partner drugs in the national treatment regimens AL and AS/AQ, increased risk of treatment failure and decreased susceptibility to lumefantrine in vitro are associated with N86, 184F, D1246, K76 alleles of *Pfmdr-1* [11, 13, 14]. AQ resistance is associated with *Pfmdr-1* alleles 86Y, Y184 and 1246Y; *P. falciparum* chloroquine resistance gene (*Pfcrt*) alleles 76T and SVMNT while CQ resistance is associated with 86Y and 76T alleles of *Pfmdr-1* and *Pfcrt* genes respectively [13, 15]. Over the years, changes in malaria treatment practices have resulted in changes in the above markers of resistance. This study aimed at assessing the prevalence of *Pfmdr-1* N86Y and D1246Y polymorphisms in *P. falciparum* isolates collected from malaria outpatients at Lira Regional Referral Hospital (LRRH), Uganda. We observed a high prevalence of wild type alleles N86 and D1246 and low prevalence of the mutant type alleles 86Y and 1246Y, which could translate to a decreased AL sensitivity in the region.

## Main text

### Methods

#### Study sites and population

This study was conducted in Lira regional referral hospital (LRRH), Lira district, Northern Uganda (1°21′N 02°42″N and 32°51″E 34°15″E), a high-endemic malaria area, located approximately 340 km north of Kampala, covering an area of approximately 1326 km^2^. Malaria is present throughout the year, with seasonal peaks of transmission coinciding with the rains (March–May and September–December). LRRH is the only regional referral facility in the sub-region, offering both specialized clinical cares, including HIV/AIDS and general clinical services. The study population consisted of febrile malaria clients attending the antiretroviral therapy (ART, 251 patients) and out patients' department (OPD, 520) clinics. Patients diagnosed with *P.* *falciparum* malaria between April and July 2015 based on Giemsa-stained thick and thin blood films were included in the study after obtaining informed consent. Inclusion criteria were individuals who presented with fevers and had taken anti-malarial drug ACT at least once during the 12 months prior to the study.

#### Sample collection

Fingerpick samples of blood spots were collected on chromatography filter paper (Lasec, Munktell TFN), air-dried, labeled and stored at − 80 °C in plastic envelopes containing silica gel until further processing. *Plasmodium* *falciparum* microscopically positive samples were used for this study and all patients diagnosed to have malaria infections were treated with artemether-lumefantrine (Coartem).

#### DNA extraction, PCR and RFLP assays

*Plasmodium falciparum* DNA was extracted from all filter paper dried blood samples using TRIzol reagent LS (Epigenetics USA) as per manufacturer’s protocol. Outer and nested PCR were performed for codons 86 and 1246 of *Pfmdr-1* on all microscopically positive samples following the protocol developed by Humphreys et al. [16], followed by restriction length polymorphism (RFLP) digestion using restriction enzymes as previously described by Thomsen et al. [17].

#### Statistical analysis

Data was analyzed using descriptive statistics in STATA version 12 (StataCorp, College Station, TX, USA). The count of samples with mutant and wild type alleles were used to generate prevalence of the *Pfmdr-1* N86Y and D1246Y alleles.

### Results

#### Prevalence of the SNPs in the pfmdr-1 gene

Out of the 200 microscopically positive samples screened for *Pfmdr-1* SNPs polymorphism (22 from ART and 178 from OPD clinics), 29.5% (n = 59/200) and 27.5% (n = 55/200) contained the outer codons c86/c184 and c1246, respectively. 84.7% (n = 50/59) and 94.5% (n = 52/55) of these samples were genotyped at codons 86 and 1246 with the elimination samples which did not amplify in the nested PCR. Our results indicate a 90% (n = 45/50) prevalence of the wild type 86N allele compared to 10% (n = 5/50) of the mutant 86Y allele (Table 1). Similarly, we observed a 92.3% (n = 48/52) prevalence of wild 1246 alleles compared to 7.7% (n = 4/52) of the mutant alleles in the study population (Table 1).

**Table 1:** Prevalence of *Pfmdr-1* polymorphisms among isolates from *P. falciparum* patients from LRRH, Uganda

SNPs codon	*Pfmdr-1* alleles	Examined	Positive	Prevalence %
Codon 1246	Wild (1246D)	52	48	92.3
	Mutant (1246Y)	52	4	7.7
Codon 86	Wild (N86)	50	45	90.0
	Mutant (86Y)	50	5	10.0

*SNPs*: single nucleotide polymorphism, *Pfmdr-1*: *P. falciparum* multidrug resistance 1 gene

### Discussion

We examined the prevalence of *Pfmdr-1* N86Y and D1246Y genotypes associated with altered drug sensitivity in febrile outpatients at LRRH utilising AL, the national treatment regimen. The prevalence of the wild type N86 and D1246 alleles were high in comparison to the mutant 86Y and 1246Y alleles (Table 1). The widespread use of AL to treat malaria has increased both the prevalence and frequency of *Pfmdr-1* wild alleles N86 and D1246, with a decrease in the sensitivity to lumefantrine and increased sensitivity to aminoquinolines [18]. Our results correlate with the findings of increased prevalence of wild type N86 and D1246 polymorphisms associated with treatment failures and decreased sensitivity to lumefantrine in Tororo, Uganda [19, 20]. Like in our study, a previous study by Okombo [21] in the neighbouring Kenya also observed a significant increase in the prevalence of wild type pfmdr1 N86/Y184/D1246 haplotype and a corresponding decline of the mutant pfmdr1 86Y/184Y/1246Y after 19 years (1995–2013) of chloroquine removal. This finding and ours highlight the importance of continued monitoring and characterization of parasite genotypes as a way to audit the therapeutic efficacy of drugs in clinical use and those previously withdrawn. The recurrent malaria infections prior to treatment with AL are associated with decreased lumefantrine sensitivity, and increased N86 genotypes could suggest significant risk factors for recrudescence in patients treated with AL [11, 18]. Interestingly, AL and AS/AQ exert opposing selective effects on the SNPs in *Pfcrt* and *Pfmdr-1* genes [11].

By year 2000, the CQ resistance in one of the eastern districts in Uganda, were over 90% [20]. In this study we found low prevalence of the mutant 86Y and 1246Y alleles which corroborates with previous findings of a gradual decrease in mutant 86Y and 1246Y genotypes in Uganda in between 2003 and 2012, following the withdrawal of CQ [20]. Prior to the withdrawal of CQ in Uganda, the CQ parasitological resistance levels ranged from < 5 to > 50% [22]. The low mutant of 86Y and 1246Y genotypes could have resulted from the decreased use of CQ and widespread use of AL as the standard anti-malarial drug in LRRH. A decreasing CQ exposure, and increased lumefantrine exposure selects for wild type sequences at these alleles [16, 23, 24]. Additionally, the low scale use of the AS/AQ, alternative first line drug for treatment of uncomplicated malaria could partly explain the low prevalence of the mutant alleles. Taken together, the current study suggests a prevalence rate of wild type alleles 1246D and N86 of about 90% compared with other studies in the region, which by 2012 was about 50% [20], suggesting high risk of treatment failures by AL. However, thus far, treatment of severe malaria in children below 20kg with intravenous AL was effective [25] indicating that, drug resistance to AL have not spread in the area.

In conclusion, our results demonstrated a high prevalence of wild type alleles N86 and D1246 that are likely to mediate decreased sensitivity to artemether-lumefantrine in the Lira RRH outpatients’ clinics. However, the mutant 86Y and 1246Y alleles showed a low prevalence owing to the decreased use of CQ and low use of AS/AQ, the alternative first line regimen in Uganda. A sustained success in malaria control is strongly dependent on the continued effectiveness of treatment using ACT, thus the need for continued countrywide monitoring of the *Pfmdr-1* SNPs.

## Limitations

We acknowledge limitations in the study including;

- The small sampling size involving only 771 out patients at LRRH participating in this study and small number of the resistance mediating polymorphism was considered.
- The possibility of sampling bias as a *P. falciparum* specific rapid kit was used for screening and existence of HRP2 and HRP3 mutant parasites.
- Low parasitemia could have led to potential false negative results especially in semi immune populations in the study area.
- The Afl-III enzyme used in genotyping could not recognize and digest both 86Y and 86F, and thereby unable to differentiate between them. However, the 86F genotype has only been reported in one study from Swaziland and not in Uganda or East Africa [26] thus all digestions with Afl-III were considered 86Y isolates.

## Abbreviations

ACT: artemisinin combination therapy
AL: artemether-lumefantrine
ART: anti-retroviral therapy
AQ: amodiaquine
DNA: deoxyribonucleic acid
IRB: Institutional review board
LRRH: Lira Regional Referral Hospital
PCR: polymerase chain reaction
*Pfcrt*: *Plasmodium falciparum* chloroquine resistant gene
*Pfmdr-1*: *Plasmodium falciparum* multi drug resistant gene-1
RFLP: restricted fragment length polymorphism
SNPs: single nucleotide polymorphisms
